# Formulation and Characterization of Antimicrobial Edible Films Based on Whey Protein Isolate and Tarragon Essential Oil

**DOI:** 10.3390/polym12081748

**Published:** 2020-08-05

**Authors:** Maria-Ioana Socaciu, Melinda Fogarasi, Cristina Anamaria Semeniuc, Sonia Ancuţa Socaci, Mihaela Ancuţa Rotar, Vlad Mureşan, Oana Lelia Pop, Dan Cristian Vodnar

**Affiliations:** 1 Department of Food Science, University of Agricultural Sciences and Veterinary Medicine Cluj-Napoca, 3-5 Mănăştur St., 400372 Cluj-Napoca, Romania; maria-ioana.socaciu@usamvcluj.ro (M.-I.S.); sonia.socaci@usamvcluj.ro (S.A.S.); anca.rotar@usamvcluj.ro (M.A.R.); oana.pop@usamvcluj.ro (O.L.P.); 2 Department of Food Engineering, University of Agricultural Sciences and Veterinary Medicine Cluj-Napoca, 3-5 Mănăştur St., 400372 Cluj-Napoca, Romania; melinda.fogarasi@usamvcluj.ro (M.F.); vlad.muresan@usamvcluj.ro (V.M.)

**Keywords:** edible films, whey protein isolate, tarragon essential oil, heat treatment, antimicrobial properties, physical properties, mechanical properties

## Abstract

The effects of heat treatment and the addition of tarragon essential oil on physical and mechanical properties of films prepared with 5% whey protein isolate (WPI) and 5% glycerol were investigated in this study. Heat treatment of the film-forming solution caused increases in thickness, moisture content, swelling degree, water vapor permeability (WVP), b*-value, ΔE*-value, transmittance values in the 200–300-nm region, transparency, and puncture resistance of the film, but decreases in water solubility, L*-value, a*-value, transmittance values in the 350–800-nm region, and puncture deformation. When incorporated with tarragon essential oil, heat-treated films have the potential to be used as antimicrobial food packaging. The addition of tarragon essential oil in film-forming solution caused increases in moisture content, solubility in water, WVP, a*-value, b*-value, ΔE*-value, and transparency of the film; decreases in transmittance values in the range of 600–800 nm; and variations in swelling degree, L*-value, transmittance values in the range of 300–550 nm, puncture resistance, and puncture deformation. Nevertheless, different tendencies were noticed in UNT (untreated) and HT (heat-treated) films with regards to transparency, light transmittance, puncture resistance, and puncture deformation. Based on these findings, HT films show improved physical and mechanical properties and, therefore, are more suitable for food-packaging applications.

## 1. Introduction

In recent years, research has focused on the development of edible films and coatings with antimicrobial activity to control microorganisms that can cause food spoilage or food poisoning [1]. Edible films and coatings have many advantages such as biodegradability, edibility, bio-compatibility, aesthetic appearance, ability to resist physical stress, and barrier properties (permeability to moisture, oxygen, aroma, and oil) [2,3].

Essential oils are among the active agents used to enhance the functionality of edible films. It is well known essential oils possess antioxidant, antibacterial, and antifungal properties and that their chemical compounds are responsible for these [1,4,5]. Several studies have reported that essential oil of tarragon exhibits antioxidant activity [6,7,8,9] and is efficient against various food-related microorganisms such as *Bacillus subtilis* [10], *Escherichia coli* [7,10], *Pseudomonas aeruginosa* [10], *Staphylococcus aureus* [7,8,10], *Listeria monocytogenes* [8], *Proteus vulgaris* [10], *Alcaligenes faecalis* [8], *Shigella dysenteriae* [8], *Aerobacter aerogenes* [10], *Candida albicans* [7,11], *Saccharomyces cerevisiae* var. *chevalieri* [11], and *Aspergillus niger* [6,7]. The essential oil content in the aerial part of tarragon plants ranges between 0.15 and 3.1%. Major components of the essential oil of tarragon differ significantly depending on the origin of the material. Essential oils extracted from Russian tarragon have been reported to be terpinen-4-ol (up to 41.34%), sabinene (up to 39%), and elemicin (up to 57%). In essential oils extracted from French tarragon, estragole (up to 74%) was found to be the main compound [12].

Preparation of edible films and coatings are based on polysaccharide, protein, or lipid polymers [13]. Compared to polysaccharides and lipids, protein-based polymers are the most useful due to impressive gas barrier properties; the oxygen permeability of soy protein-based films is 260, 500, 540, and 670 times lower than that of low-density methyl cellulose, polyethylene, starch, and pectin [14]. Whey proteins can produce films with excellent barrier properties to oxygen, aroma, and oil at low-to-intermediate relative humidity and also with adequate mechanical properties that provide them durability when used as coatings or films for packaging applications [14,15]. Whey protein isolate (WPI) is the whey protein product with the highest protein content, more than 90% water-soluble milk proteins [16]. It is produced from whey protein concentrate (WPC) through demineralization by ion exchange [15]. Native whey proteins do not exhibit good adhesive properties due to their low molecular weights and compact globular structures [17]. Heat denaturation above 65 °C opens the *β*-lactoglobulin globular structure, exposes sulfhydryl and hydrophobic groups, and thus induces oxidation of free sulfhydryls, disulfide bond interchange, and hydrophobic bonding. These reactions result in the formation of water-insoluble edible films, with good adhesive properties [17,18]. The making of protein-based films generally requires the incorporation of a minimal content of plasticizer, 3%, to reduce the brittleness [19,20]. The addition of a plasticizer in the denatured film solutions improves the flexibility of films but increases the water vapor permeability (WVP) [21]. The most commonly used plasticizer for the preparation of protein-based films is glycerol since it is miscible in most proteins [22].

In light of the preceding considerations, this work aimed to gather a set of data to support the comparative characterization of whey protein isolate (WPI)-based edible films obtained from untreated (UNT) and heat-treated (HT) film-forming solutions with different levels of incorporated tarragon essential oil (0.5, 1.0, 1.5, 2.0, and 2.5%, *w*/*w*). The current study focused on (1) the characterization of tarragon essential oil by determination of its total phenolic content, volatile constituents, antioxidant capacity, and antibacterial activity against *Staphylococcus aureus*, *Escherichia coli*, *Salmonella enteritidis*, and *Listeria monocytogenes*, and (2) the characterization of films by determination of their thickness, moisture content, swelling degree, solubility in water, water vapor permeability (WVP), color, light transmittance, transparency, puncture resistance (PR), and puncture deformation (PD).

## 2. Materials and Methods

### 2.1. Plant Material and Essential Oil Extraction

For the extraction of essential oil, commercially available dried leaves of tarragon were purchased from a company that markets food ingredients (Solina Group, Alba Iulia, Romania, http://www.solina-group.ro). The essential oil of tarragon was obtained by hydrodistillation using a Clevenger-type apparatus (S.C. Energo-Metr S.R.L., Odorheiu Secuiesc, Romania), 50 g of dried leaves being boiled for 3 h with 750 mL distilled water. The essential oil of tarragon was dried over anhydrous sodium sulphate and stored at 4 °C prior to analysis. The extraction yield was calculated as the volume of essential oil (mL) per dried leaves’ weight (g) and multiplied by 100 [5].

#### 2.1.1. ITEX/GC-MS Analysis of Tarragon Essential Oil

The qualitative ITEX/GC-MS (in-tube extraction coupled with gas chromatography-mass spectrometry) analysis of volatile constituents was carried out as described in our previous report [5]. A sample of 1 µL volume was measured into a sealed-cap headspace vial (20 mL) and maintained on continuous agitation at 60 °C for 10 min. The adsorption (5 strokes) of volatile constituents from the gaseous phase of tarragon essential oil was performed by a Combi PAL AOC-5000 autosampler (CTC Analytics, Zwingen, Switzerland) with a headspace syringe ITEX-II equipped with a microtrap (ITEX-2TrapTXTA, Tenax TA 80/100 mesh, Switzerland). The analytes were released by thermal desorption into the injection port of the GC-MSQP2010 system (Shimadzu, Kyoto, Japan); the microtrap was then flash-heated with N_2_.

A Zebron ZB-5 ms capillary column (30 m × 0.25 mm i.d. × 0.25 μm film thickness; Phenomenex, Torrance, CA, USA) was used for the chromatographic separation of volatile constituents. The column oven temperature program was set as follows: From 40 °C (kept at this temperature for 2 min) to 160 °C at 4 °C/min, then raised to 240 °C at 15 °C/min (kept at this temperature for 5 min). The temperature of the ion source, injector, and interface was set at 250 °C. Helium was used as the carrier gas at a flow rate of 1 mL/min. The split ratio was 1:300. The ion trap mass spectrometer was operated in EI-MS mode; the acquisition mode was set in the range of 40–650 *m*/*z*.

The volatile compounds were tentatively identified by comparing their mass spectra with those in the NIST27 and NIST147 libraries (considering a minimum similarity of 85%) and by retention indices obtained from www.pherobase.com [23] or www.flavornet.org (for columns with a similar stationary phase to ZB-5 ms) [24]. The results were expressed as the relative percentage of each compound from the total ion chromatograms (TIC) area (100%).

#### 2.1.2. Determination of Total Phenolic Content

The total phenolic content was measured using the method described by Semeniuc et al. (2018) [5]. One hundred microliters of the test sample (dilution of tarragon essential oil with methanol, 1:50 (*v*/*v*)) were transferred into a 16-mL glass bottle with a rubber stopper. Then, 6 mL of distilled water and 0.5 mL of 2 N Folin-Ciocalteu phenol reagent were added and immediately vortexed (Vortex V-1 Plus, Biosan Ltd., Riga, Latvia). After 4 min, 1.5 mL of 0.71 M sodium carbonate aqueous solution and 1.9 mL of distilled water were added to the mixture. The incubation of the test sample was carried out in the dark at room temperature for 2 h. The absorbance value was read at 725 nm against the blank sample using a double-beam UV-VIS spectrophotometer (PharmaSpec UV-1700, Shimadzu, Kyoto, Japan). The blank sample was prepared with methanol and treated identically to the test sample. Three readings per sample were taken. The result was expressed in mg gallic acid equivalents (GAE) 100 µL^−^^1^ EO.

#### 2.1.3. Determination of Antioxidant Capacity

The Trolox equivalent antioxidant capacity assay was used to measure the antioxidant capacity of tarragon essential oil; it was performed according to the method described by Semeniuc et al. (2018) [5]. The stock solution was prepared by mixing equal volumes of 7.4 mM ABTS aqueous solution and 2.6 mM potassium persulfate aqueous solution and allowing them to react for 12 h at room temperature in the dark. The working solution was prepared by mixing 1 mL of stock solution with 60 mL of methanol to obtain an absorbance of 1.1 ± 0.02 units at 734 nm.

One hundred fifty microliters of the test sample (dilution of tarragon essential oil with methanol, 1:100 (*v*/*v*)) were transferred into a 16-mL glass bottle with a rubber stopper. Then 2850 μL of ABTS working solution were added and vortexed (Vortex V-1 Plus, Biosan Ltd., Riga, Latvia). The incubation of the test sample was carried out in the dark at room temperature for 2 h. The absorbance value was read at 734 nm against methanol using a double-beam UV-VIS spectrophotometer (PharmaSpec UV-1700, Shimadzu, Kyoto, Japan). The blank sample was prepared with methanol and treated identically to the test sample. Three readings per sample were taken. The absorbance value of the test sample was extracted from the absorbance value of the blank sample; the final result was expressed in µM Trolox equivalent (TE) mL^−^^1^ EO.

### 2.2. Bacterial Strains

The following microorganisms were tested: *Staphylococcus aureus* (ATCC 25923, Microbiologics Inc., St. Cloud, MN, USA), *Escherichia coli* (ATCC 25922, Microbiologics Inc., St. Cloud, MN, USA), *Salmonella enteritidis* (ATCC 13076, Microbiologics Inc., St. Cloud, MN, USA), and *Listeria monocytogenes* (ATCC 19114, Microbiologics Inc., St. Cloud, MN, USA). Each strain was grown into a test tube containing 10 mL sterile nutrient broth (Oxoid Ltd., Basingstoke, Hampshire, UK) at 37 °C for 24 h in the case of *S. aureus*, *E. coli*, and *S. enteritidis* and at 37 °C for 48 h in the case of *L. monocytogenes*. The purity of the inoculum solution was confirmed by plating on appropriate selective media and microscopic examination of the Gram-stained smear (Optika microscope, B-252, M.A.D. Apparecchiature Scientifiche, Milan, Italy). A loopful of the inoculum solution was transferred by streaking onto a selective medium: (1) Baird-Parker agar base supplemented with Egg Yolk Tellurite Emulsion (Oxoid Ltd., Basingstoke, Hampshire, UK) for *S. aureus*, (2) TBX agar (Oxoid Ltd., Basingstoke, Hampshire, UK) for *E. coli*, (3) XLD agar (Oxoid Ltd., Basingstoke, Hampshire, UK) for *S. enteritidis*, and (4) Palcam agar base (Oxoid Ltd., Basingstoke, Hampshire, UK) with added Palcam selective supplement for *L. monocytogenes*. Plates were then incubated at 37 °C for 24 h (in the case of *S. aureus*, *E. coli*, and *S. enteritidis*) and 48 h (in the case of *L. monocytogenes*). Bacterial morphology was confirmed by optical microscopy. Several colonies were collected with a sterile inoculating loop, transferred into sterile saline solution (8.5 g L^−1^), and adjusted to match the turbidity of a McFarland 0.5 standard (1.5 × 10^8^ CFU mL^−1^) [25]. Then, three serial 10-fold dilutions (10^7^, 10^6^, and 10^5^ CFU mL^−1^) were prepared using the sterile saline solution as diluent.

#### 2.2.1. Kirby-Bauer Disk Diffusion Test

The test was performed according to the method described by Semeniuc et al. (2017) [26]. The tarragon essential oil was assessed against all bacteria using 9-mm sterile paper disks (ANTF-009-1K0, PRAT DUMAS, France). Gentamicin (0.4 mg mL^−^^1^ in saline solution) was used as a positive control. One hundred microliters of the inoculum solution (1.5 × 10^8^ CFU mL^−^^1^) were dispersed over the entire surface of the Mueller-Hinton agar plate (Sifin Diagnostics GmbH, Berlin, Germany) using a Drigalski spatula. A sterile paper disk was placed in the middle of a Petri dish, and 40 µL of tarragon essential oil were released on it. Plates were then incubated at 37 °C for 24 h (in the case of *S. aureus*, *E. coli*, and *S. enteritidis*) and 48 h (in the case of *L. monocytogenes*). A digital caliper (Powerfix Profi Z22855, Milomex Ltd., Bedfordshire, UK) was used to measure the inhibition zone diameter. Three replicates were run for each sample. The result was expressed in millimeters.

#### 2.2.2. Minimum Inhibitory Concentration (MIC) Test 

The test was performed according to the method described by Semeniuc et al. (2018) [5]. Eight parts 50% ethanol solution and one part Tween 80 were mixed with one part of tarragon essential oil. Sterile nutrient broth (100 µL) and the mixture of essential oil (100 µL) were added into the first well of a 96-well microtiter plate. Serial 11-fold dilutions were obtained by pipetting 100 µL from well to well (on the same row), while 100 µL were discarded from the last well of the row. Next, a volume of 10 µL inoculum solution (1.5 × 10^5^ CFU mL^−^^1^) was added to each well. Concentrations ranging from 0.01 to 47.62 µL EO mL^−^^1^ were thus reached. For the negative control, eight parts 50% ethanol solution and one part Tween 80 were mixed with one part saline solution, while gentamicin (0.4 mg mL^−^^1^ in saline solution) was considered the positive control. Microplates were incubated at 37 °C for 21 h (in the case of *S. aureus*, *E. coli*, and *S. enteritidis*) and 45 h (in the case of *L. monocytogenes*). A volume of 20 μL resazurin aqueous solution (0.2 mg mL^−^^1^) was added to each well. Microplates were subsequently incubated at 37 °C for 2 h (in the case of *S. aureus*, *E. coli*, and *S. enteritidis*) and 23 h (in the case of *L. monocytogenes*). The lowest concentration that retained the blue color was considered the concentration that completely inhibited bacterial growth (MIC). Three replicates were run for each sample. The result was expressed in µL EO mL^−^^1^ and µg gentamicin (GE) mL^−^^1^.

#### 2.2.3. Minimum Bactericidal Concentration (MBC) Test 

The test was performed according to the method described by Semeniuc et al. (2018) [5]. To determine the minimum bactericidal concentration (MBC), 10 µL of the dilution representing the MIC and the more concentrated two dilutions were plated on the appropriate selective media (Baird-Parker agar base supplemented with Egg Yolk Tellurite Emulsion for *S. aureus*, TBX agar for E. coli, XLD agar for *S. enteritidis*, and Palcam agar base with added Palcam selective supplement for *L. monocytogenes*) using a Drigalski spatula and incubated for 48 h at 37 °C. A colony counter (Colony Star 8500, Funke Gerber, Berlin, Germany) was used to determine the relative number of bacterial colonies. The lowest concentration of the antimicrobial agent causing negative growth (fewer than three colonies) was considered the concentration that completely killed bacteria (MBC). Three replicates were run for each sample. The result was expressed in µL EO mL^−^^1^.

### 2.3. Films’ Preparation

UNT films were prepared from native WPI solutions and HT films from heat-denatured WPI solutions. Film-forming solutions were obtained by dissolving 5% (*w*/*w*) WPI in distilled water, according to the modified protocol described by Badr et al. (2014) [27]. Glycerol was added as a plasticizer in filmogenic solutions, at a concentration of 5% (*w*/*w*). The WPI (Prolacta 95 LL Instant, Lactalis, France) was purchased from REDIS C.O. S.R. (Bucharest, Romania), and the glycerol from Chempur (Piekary Śląskie, Poland).

For HT films, solutions were subsequently heated for 30 min. at 90 ± 2 °C while being continuously stirred using a magnetic stirrer with heating (MSH-300, Biosan Ltd., Riga, Latvia). Heated solutions were then cooled at room temperature for 1.5 h and filtered to remove any air incorporated during stirring.

The essential oil of tarragon was added both in native WPI and heat-denatured WPI solutions in different amounts, to obtain formulations of 0.5% (F0.5), 1.0% (F1), 1.5% (F1.5), 2.0% (F2), and 2.5% (F2.5). Solutions were then homogenized at 23,000 rpm for 2.5 min using a laboratory dispenser (T 18 digital Ultra-Turrax, IKA-Werke GmbH & Co. KG, Staufen, Germany). The final film-forming solutions were poured (4.8 g) into disposable weighing dishes (43 × 13 mm) and then dried in an oven (Digitheat, J.P. Selecta S.A., Barcelona, Spain) at 37 °C (42 h). Once formed, the films were peeled off and stored individually in airtight containers, at room temperature, until analysis.

#### 2.3.1. Measurement of Thickness

The thickness (mm) of each film formulation was measured at 24 random points using a digital caliper (Powerfix Profi Z22855, Milomex Ltd., Bedfordshire, UK).

#### 2.3.2. Determination of Moisture Content

The moisture content was determined using the oven-drying method by the differential weighing of the film sample before and after drying. The film specimen was cut approximately into equal quarters; each quarter was dried in an oven (Digitheat, J.P. Selecta S.A., Barcelona, Spain) at 105 °C until constant weight. The moisture content was calculated using the following Formula (1):

(1) $$Moisture\;Content\;\left(\%\right)=\frac{w_{i}-w_{d}}{w_{i}}\times 100$$

where $w_{i}$ is the initial weight of the film sample (g) and $w_{d}$ is the oven-dry weight of the film sample (g). Four replicates were run for each film formulation.

#### 2.3.3. Determination of Swelling Degree and Solubility in Water

These parameters were determined according to a slightly modified method of Wang et al. (2010) [28]. The film specimen was cut into square pieces. A piece of the film was accurately weighed (~0.1 g) into a 50-mL glass beaker, and then 40 mL of distilled water were added. After 24 h of immersion at room temperature (~25 °C), the remnant was filtered and weighed, for the determination of swelling degree or filtered and dried in an oven (Digitheat, J.P. Selecta S.A., Barcelona, Spain) at 105 °C until constant weight, for the determination of solubility in water. The swelling degree was calculated by (2) and the solubility in water by (3):

(2) $$Swelling\;Degree\;\left(\%\right)=\frac{w_{i}-w_{f}}{w_{i}}\times 100$$

(3) $$Solubility\;in\;Water\;\left(\%\right)=\frac{w_{i}-w_{d}}{w_{i}}\times 100$$

where $w_{i}$ is the initial weight of the film sample (g), $w_{f}$ is the weight of the remnant after filtration (g), and $w_{d}$ is the oven-dry weight of the remnant (g). Three replicates were run for each film formulation in both cases.

#### 2.3.4. Determination of Water Vapor Permeability (WVP)

The WVP was determined according to the method described by Ghasemlou et al. (2013), with slight modification [29]. The film was sealed on a plastic cup (commercially available), containing 5.0 g of calcium chloride anhydrous (CaCl_2_—0% RH), with an exposed area of 4.0 cm^2^. The cup was stored at room temperature in a desiccator containing a saturated sodium chloride solution (~25 °C, NaCl—60% relative humidity). The weight of the cup (w) was recorded every hour during the first 8 h, and every day for 14 days. The weight gain (Δw) was plotted as a function of time. The slope (a) of each line was calculated by linear regression; the correlation coefficient was > 0.99 at all film formulations. The WVP was calculated with the following Equation (4):

(4) $$WVP\;\left(g\;mm/m^{2}day\;kPa\right)=\frac{a\times X}{A\times \Delta p}$$

where a is the slope of the regression line, X is the film thickness (mm), A is the area of exposed film (m^2^), and Δp is the vapor pressure differential across the tested film (kilopascals). Three replicates were run for each film formulation.

#### 2.3.5. Measurement of Color

The color was measured using an NH300 portable colorimeter (3NH, Shenzhen, China) based on the CIE L*a*b* color system. The L*-value represents lightness and ranges from zero (the darkest black) to 100 (the brightest white). The a*-value represents “redness” or “greenness” and ranges from +60 for absolute red to −60 for absolute green, while b*-value represents “yellowness” or “blueness” and ranges from +60 for absolute yellow to −60 for absolute blue. Measurements were performed using a D65 illuminant with an opening of 8 mm and a 10° standard observer. The colorimeter was subjected to automatic black and white calibration. The film specimen was placed on the surface of a standard white plate ($L_{s}^{*}=90.75,\;a_{s}^{*}=-0.082,\;b_{s}^{*}=-2.71$) before measuring. The total color difference (ΔE*) was calculated by (5):

(5) $$\Delta E^{*}=\sqrt{{\left(L^{*}-L_{s}^{*}\right)}^{2}+{\left(a^{*}-a_{s}^{*}\right)}^{2}+{\left(b^{*}-b_{s}^{*}\right)}^{2}}$$

where $L^{*},\;a^{*},\;\mathrm{and}\;b^{*}$ are the color values of the film sample and $L_{s}^{*},a_{s}^{*},\;\mathrm{and}\;b_{s}^{*}$ of the standard white plate. Twelve readings were taken on each film formulation.

#### 2.3.6. Measurement of Light Transmittance and Transparency

Light transmittance percentage and transparency value were determined using a double-beam UV-VIS spectrophotometer (PharmaSpec UV-1700, Shimadzu, Kyoto, Japan) by reading the absorbance of the film sample at wavelengths between 200–800 nm. Transmittance is the percent of incident light that passes through a material sample and is determined by the effectiveness of the absorption and scattering of light by the material. Transparent material has a transmittance above 90% [30].

The film specimen was cut into 3 strips (0.7 cm × 3 cm). Each strip was placed in a quartz cuvette (100-QS, 10 mm lightpath, Hellma Analytics, Müllheim, Germany), and its absorbance read against an empty cuvette. Conversion of the absorbance value to percent transmittance was done using the Lambert-Beer Equation (6):

(6) $$Light\;Transmittance\;\left(\%\right)=antilog_{10}\;\left(2-A\right)$$

where A is the absorbance value of the film strip.

The relative transparency of film strip was measured at 600 nm, and calculated with the formula [31] (7):

(7) $$Transparency\;\left(A600/\mathrm{mm}\right)=\frac{A600}{X}$$

where A600 is the absorbance value at 600 nm and X is the film thickness (mm). Three readings were taken on each film formulation.

#### 2.3.7. Puncture Test

The mechanical properties of the film (puncture resistance (PR) and puncture deformation (PD)) were determined by the puncture test proposed by Mitrea et al. (2020), using a texture analyzer (CT3, Brookfield Engineering Laboratories Inc., Middleboro, MA, USA) [32]. The film specimen was placed in the middle of two metallic washers (commercially available), with a hole of 10 mm in diameter, and fixed between them with two clamps. The film support fixture was then placed onto the base table and fastened into position. The puncture head (a cylindrical rod of 2 mm in diameter-TA39), was set to a target distance of 5.0 mm with a speed of 0.5 mm/s. Measurements were performed in four replicates for each formulation type. To avoid any effect of thickness fluctuation, the measured values were divided by the thickness of the film and reported as N/mm for PR and as mm for PD.

### 2.4. Statistical Analysis

Data analysis was carried out using Minitab statistical software (version 16.1.0; LEAD Technologies, Inc., Charlotte, NC, USA). The effects of heat treatment of the film-forming solution and the addition of tarragon essential oil on thickness, moisture content, swelling degree, solubility in water, WVP, color, light transmittance, transparency, PR, and PD of the film were analyzed using analysis of variance (ANOVA) by applying a general linear model (see Tables S1–S4 in Supplementary Material). Post hoc pairwise comparisons were performed with Tukey’s test at a 95% confidence level (*p* < 0.05). Statistical significance of the effects was interpreted as follows: *p* ≥ 0.05^NS^, not significant; *p* < 0.05 *, significant; *p* < 0.01 **, very significant; *p* < 0.001 ***, extremely significant. The percentage contribution of each factor and their interaction was calculated using eta-squared (*η*^2^).

## 3. Results

### 3.1. Chemical and Biological Properties of Tarragon Essential Oil

The essential oil extracted from dried leaves of tarragon showed an extraction yield of 0.62% (*v*/*w*), total phenolic content of 2.3 ± 0.099 mg GAE 100 µL^−1^ EO, and antioxidant capacity of 31.0 ± 0.291 µM TE mL^−1^ EO.

The volatile compounds detected in tarragon essential oil by ITEX/GC-MS analysis are listed in Table 1. Sixteen compounds, representing 100% of the total detected constituents, were identified in the essential oil of tarragon and grouped based on their chemical structure into four classes (monoterpene hydrocarbons-C1, phenylpropanoids-C2, oxygenated monoterpenes-C3, and aliphatic aldehydes-C4). The most abundant constituents were monoterpene hydrocarbons (96.66%), followed by phenylpropanoids (2.18%), oxygenated monoterpenes (0.99%), and aliphatic aldehydes (0.18%). The major components identified in tarragon essential oil were sabinene (74.98%), *γ*-terpinene (3.80%), D-limonene (3.69%), *β*-myrcene (3.38%), 4-carene (2.58%), and *α*-phellandrene (2.27%), all of them belonging to the monoterpene hydrocarbons’ class.

Results of the Kirby-Bauer disk diffusion test are presented in Table 2. The essential oil of tarragon exhibited the highest inhibitory effect against *S. enteritidis*, followed by *S. aureus*, *E. coli*, and *L. monocytogenes*.

Table 3 summarizes the results of MIC and MBC tests. The lower the MIC/MBC value, the higher the antibacterial activity of essential oil [26]. The essential oil of tarragon revealed both bacteriostatic and bactericidal properties as follows: Against *E. coli* > *L. monocytogenes* > *S. enteritidis* > *S. aureus*.

### 3.2. Physical and Mechanical Properties of Films

#### 3.2.1. Films’ Appearance

**Figure 1:**
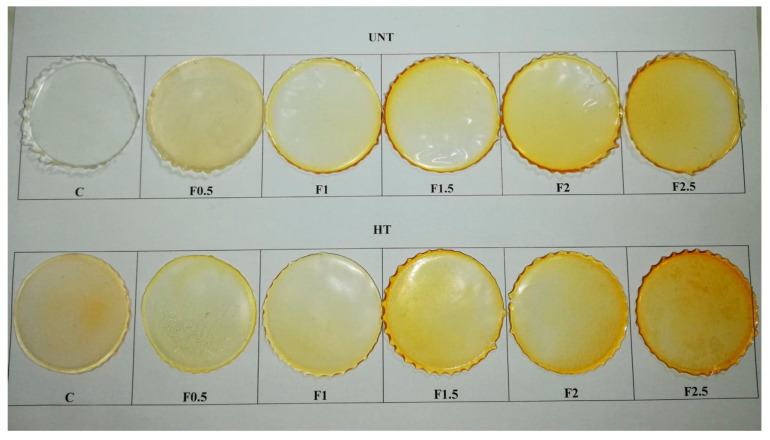
Surface images of UNT and HT formulations. C-control film; F0.5-film with 0.5% tarragon essential oil; F1-film with 1% tarragon essential oil; F1.5-film with 1.5% tarragon essential oil; F2-film with 2% tarragon essential oil; F2.5-film with 2.5% tarragon essential oil.

Figure 1 shows the surface images of UNT and HT formulations. Both UNT and HT films were flexible, yellowish, and semi-transparent; the changes in color and transparency with the amount of added tarragon essential oil are discussed in the Section 3.2.6 and Section 3.2.7.

Surfaces of films were smooth, with no evidence of pores, cracks, or fissures. The appearance of the two sides of the film was different for both UNT and HT films; the film side facing the casting dish was dull, while the other was shiny. This indicates some phase separation during drying, visible on UNT films as very fine oil droplets at the surface. On HT films, formulations F1.5, F2, and F2.5 showed discontinuities, probably due to oil droplet flocculation.

HT films were easily detached by the casting dishes, while UNT films were rather sticky.

**Figure 2:**
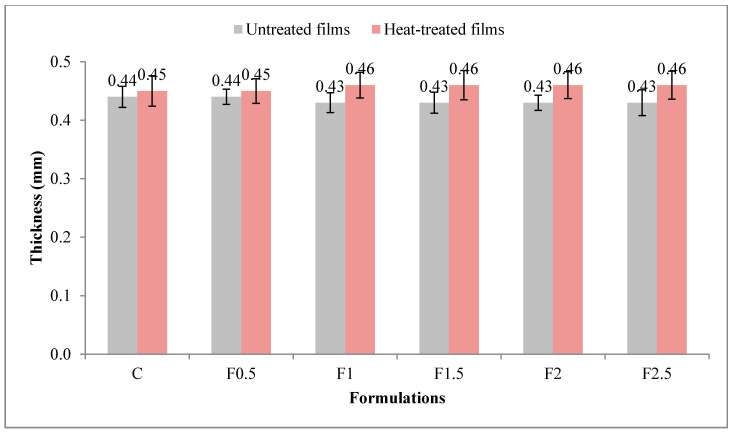
Variation in film thickness with heat treatment and formulation. C-control film; F0.5-film with 0.5% tarragon essential oil; F1-film with 1% tarragon essential oil; F1.5-film with 1.5% tarragon essential oil; F2-film with 2% tarragon essential oil; F2.5-film with 2.5% tarragon essential oil. Values are expressed as mean ± standard deviation of 24 replicates.

Values obtained for the thickness of UNT and HT formulations are shown in Figure 2. The heat treatment of the film-forming solution had a significant effect (*p* < 0.001 ***) on film thickness, with a contribution of 21.8%, the HT film being thicker than the UNT one (see Table S1 in Supplementary Material). In contrast, the addition of tarragon essential oil in a concentration of up to 2.5% did not have a significant effect (*p* > 0.05^NS^; contribution of 1%) on the film thickness. In UNT films, thickness ranged between 0.43 and 0.44 mm, while in HT ones, between 0.45 and 0.46 mm.

#### 3.2.2. Moisture Content

**Figure 3:**
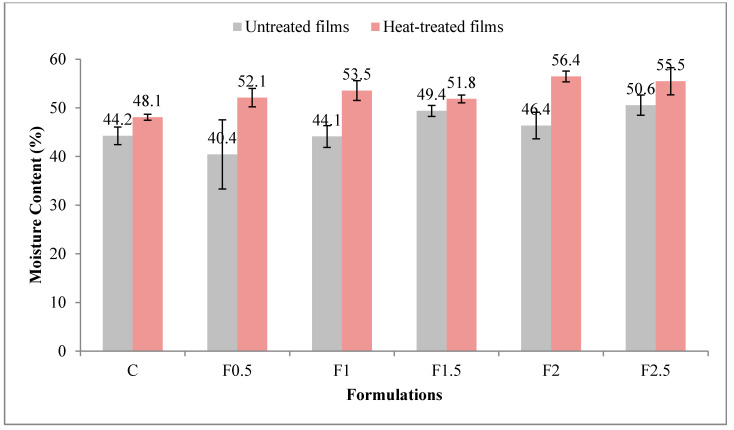
Variation in moisture content with heat treatment and formulation. C-control film; F0.5-film with 0.5% tarragon essential oil; F1-film with 1% tarragon essential oil; F1.5-film with 1.5% tarragon essential oil; F2-film with 2% tarragon essential oil; F2.5-film with 2.5% tarragon essential oil. Values are expressed as mean ± standard deviation of four replicates.

This parameter indicates the total void volume occupied by water molecules in the network microstructure of the film [28]. Figure 3 shows moisture-content values for UNT and HT films. The moisture content was significantly higher (*p* < 0.001 ***) in the HT film compared to the UNT one, the contribution of the heat treatment of the film-forming solution being 45.2% (see Table S1 in Supplementary Material). The addition of tarragon essential oil also significantly influenced (*p* < 0.001 ***) the moisture content of the film, with a contribution of 23.7%, resulting in its increase with the added amount. The moisture content showed values between 40.4 and 50.6% in UNT films and between 48.1 and 56.4% in HT ones.

#### 3.2.3. Swelling Degree

**Figure 4:**
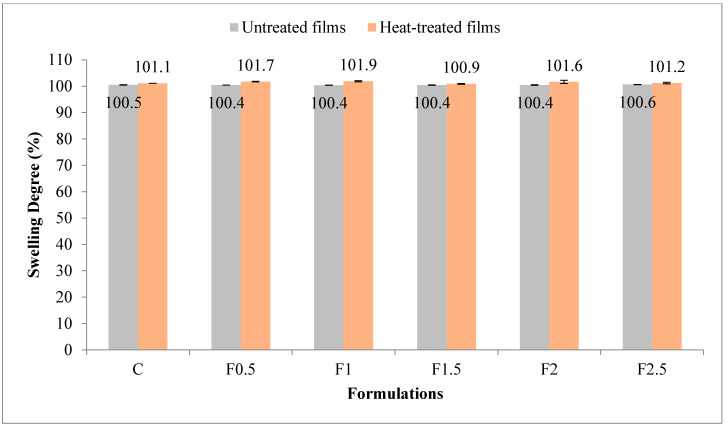
Variation in swelling degree with heat treatment and formulation. C-control film; F0.5-film with 0.5% tarragon essential oil; F1-film with 1% tarragon essential oil; F1.5-film with 1.5% tarragon essential oil; F2-film with 2% tarragon essential oil; F2.5-film with 2.5% tarragon essential oil. Values are expressed as mean ± standard deviation of three replicates.

Swelling is an undesirable property for an edible film, especially if destined for packaging of food with high moisture content [33]. Figure 4 illustrates the values for swelling degree in UNT and HT films. The swelling degree was significantly affected (*p* < 0.001 ***) by heat treatment of the film-forming solution, with a contribution of 68.1% (see Table S1 in Supplementary Material). The value of this parameter was higher in HT film compared to UNT one. The contribution of tarragon essential oil addition was also significant (*p* < 0.01 **) but lower as a share (9.2%). The swelling degree value ranged, with the addition of tarragon essential oil, between 100.4 and 100.6% in UNT films, and between 100.9 and 101.9% in HT ones. A much higher swelling degree, approximately 350%, was found by Wang et al. (2010) in edible film prepared with 10% WPI and 5% glycerol [28].

#### 3.2.4. Solubility in Water

**Figure 5:**
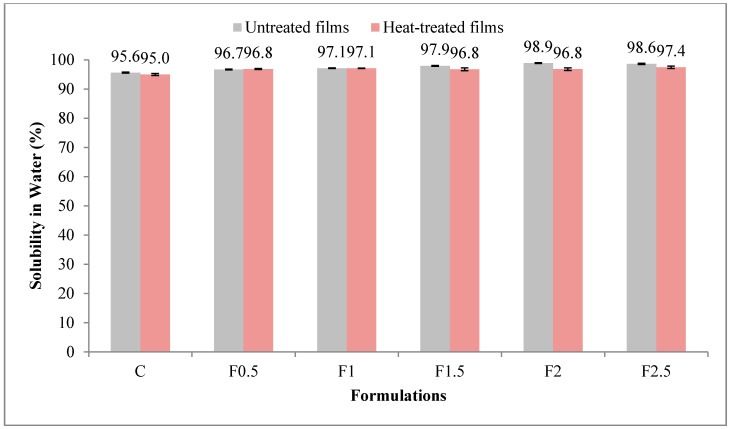
Variation of solubility in water with heat treatment and formulation. C-control film; F0.5-film with 0.5% tarragon essential oil; F1-film with 1% tarragon essential oil; F1.5-film with 1.5% tarragon essential oil; F2-film with 2% tarragon essential oil; F2.5-film with 2.5% tarragon essential oil. Values are expressed as mean ± standard deviation of three replicates.

The solubility in water is a parameter related to the hydrophilicity of film [34]. Values obtained for the solubility in water of UNT and HT formulations are shown in Figure 5. In UNT films, solubility in water ranged from 95.6 to 98.9%, and in HT ones, from 95.0 to 97.4%. Ramos et al. (2012) reported a lower value of solubility in water, 67.6%, in their edible film prepared with 10% WPI and 5% glycerol [35].

The solubility in water was significantly lower (*p* < 0.001 ***) in the HT film as opposed to UNT one, indicating lower hydrophilicity (see Table S1 in Supplementary Material); an explanation may be that protein agglomerates have blocked the micropaths in the network microstructure [28]. The contribution of the thermic factor was 14.5%. The value of solubility in water significantly increased (*p* < 0.001 ***) with the addition of tarragon essential oil, thus enhancing the film hydrophilicity, and the contribution of this factor was higher, of 68.7%.

#### 3.2.5. Water Vapor Permeability (WVP)

**Figure 6:**
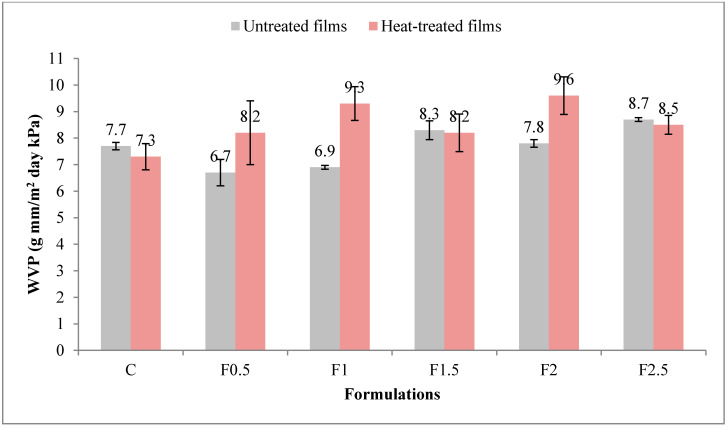
Variation in water vapor permeability (WVP) with heat treatment and formulation. C-control film; F0.5-film with 0.5% tarragon essential oil; F1-film with 1% tarragon essential oil; F1.5-film with 1.5% tarragon essential oil; F2-film with 2% tarragon essential oil; F2.5-film with 2.5% tarragon essential oil. Values are expressed as mean ± standard deviation of three replicates.

The WVP of a film is directly related to the micropaths in its network microstructure [34]; the higher the WVP value, the more permeable the film is to moisture. Figure 6 displays the WVP values for both UNT and HT films. In UNT films, WVP ranged between 6.7 and 8.7 g mm/m^2^ day kPa while in HT ones, between 7.3 and 9.6 g mm/m^2^ day kPa. Comparable values of WVP were reported by Pérez-Gago et al. (1999) in edible films prepared with 5% WPI and 12% glycerol, of 5.1 g mm/m^2^ day kPa in the UNT film and 5.0 g mm/m^2^ day kPa in the HT film [36]. Higher levels of WVP were found by Wang et al. (2010) and Ramos et al. (2012) in their edible films prepared with 10% WPI and 5% glycerol, of 22.0 g mm/m^2^ day kPa and 10.1 g mm/m^2^ day kPa, respectively [28,35].

The WVP was significantly influenced (*p* < 0.01**) by heat treatment of the film-forming solution, with a contribution of 19.9% (see Table S1 in Supplementary Material). Its value was higher in HT film, thus revealing a higher permeability to moisture than the UNT film. The WVP was also significantly influenced (*p* < 0.05 *) by the addition of tarragon essential oil (contribution of 27.8%), increasing with the added amount. Consequently, the moisture barrier properties of the film were diminished.

#### 3.2.6. Color

**Figure 7:**
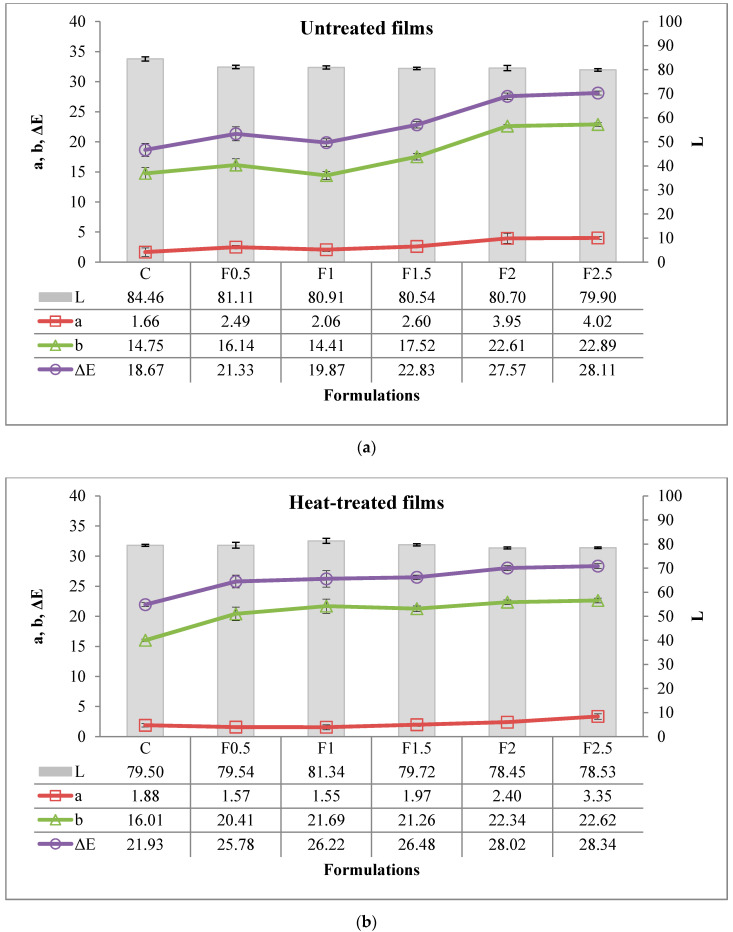
Color attributes of edible films. (**a**) Untreated, (**b**) heat-treated. C-control film; F0.5-film with 0.5% tarragon essential oil; F1-film with 1% tarragon essential oil; F1.5-film with 1.5% tarragon essential oil; F2-film with 2% tarragon essential oil; F2.5-film with 2.5% tarragon essential oil. Values are expressed as mean ± standard deviation of 12 replicates.

Color attributes (L*, a*, b*, and ΔE*) for UNT and HT films, containing various levels of tarragon essential oil, are shown in Figure 7a,b. The ΔE* provides a good measure of the color difference since it takes into account all three color parameters: Lightness (L*), red-green (a*), and yellow-blue (b*) components [31]. The heat treatment of the film-forming solution had a significant effect (*p* < 0.001 ***) on the color parameters of film, with a contribution of 27.5% for L*, of 13.3% for a*, of 16.6% for b*, and of 20.2% for ΔE* (see Table S2 in Supplementary Material). Values for L* and a* were lower in HT film and values for b* and ΔE*, in UNT one. Therefore, the HT film had a darker and more yellowish color compared to UNT one but less reddish. The addition of tarragon essential oil also had a significant effect (*p* < 0.001 ***) on the color parameters of film, the contribution of this factor being higher, of 30.6% for L*, of 60.4% for a*, of 61.9% for b*, and of 65.0% for ΔE*. L*-values oscillated with the addition of tarragon essential oil and a*-, b*-, and ΔE*-values increased, resulting in a red-yellowish color of the film. In UNT films, L*-value ranged between 84.46 (C-control film) and 79.90 (F2.5), a*-value between 1.66 (C) and 4.02 (F2.5), b*-value between 14.75 (C) and 22.89 (F2.5), while ΔE*-value was between 18.67 (C) and 28.11 (F2.5). With regard to HT films, L*-value ranged between 79.50 (C) and 78.53 (F2.5), a*-value between 1.88 (C) and 3.35 (F2.5), b*-value between 16.01 (C) and 22.62 (F2.5), while ΔE*-value was between 21.93 (C) and 28.34 (F2.5).

#### 3.2.7. Light Transmittance and Transparency

The ultraviolet (UV) and light-visible (VIS) barrier properties of the film were measured at various wavelengths (in the range of 200 to 800 nm), using a double-beam UV-VIS spectrophotometer. Good barrier properties to UV and VIS light are indicated by low transmittance values in the range of 200–350 nm and 400–800 nm, respectively.

**Figure 8:**
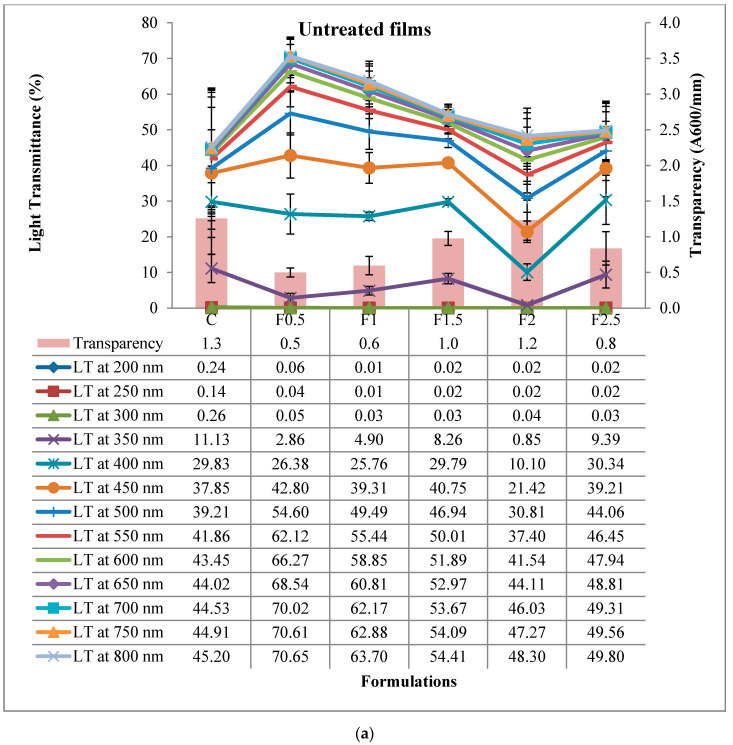

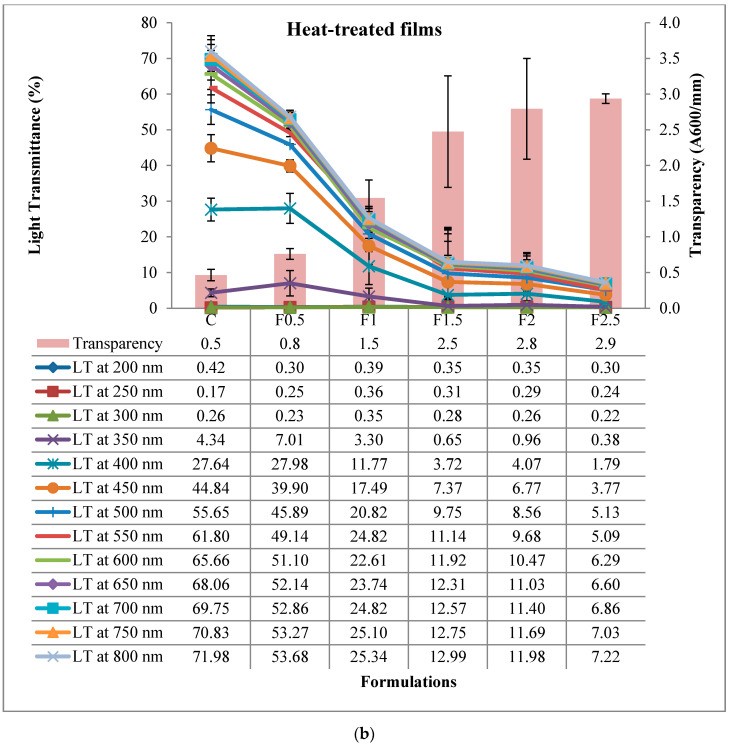
Light transmittance and transparency of edible films. (**a**) Untreated, (**b**) heat-treated. C-control film; F0.5-film with 0.5% tarragon essential oil; F1-film with 1% tarragon essential oil; F1.5-film with 1.5% tarragon essential oil; F2-film with 2% tarragon essential oil; F2.5-film with 2.5% tarragon essential oil. Values are expressed as mean ± standard deviation of three replicates for light transmittance and transparency.

Light transmittance percentages in the UV-VIS range, as well as transparency values related to UNT and HT films, are shown in Figure 8a,b. The heat treatment of the film-forming solution had a significant effect (*p* < 0.001 ***) on the transmittance of film at all analyzed wavelengths, but with different contributions: Of 66.6% at 200 nm, 71.9% at 250 nm, 58.4% at 300 nm, 19.2% at 350 nm, 28.0% at 400 nm, 28.1% at 450 nm, 28.1% at 500 nm, 28.8% at 550 nm, 30.1% at 600 nm, 30.1% at 650 nm, 30.3% at 700 nm, 30.0% at 750 nm, and 29.7% at 800 nm (see Table S3 in Supplementary Material). With few exceptions (at 200, 250, and 300 nm), the transmittance values were lower in HT film. In the UV-light domain (200–350 nm), transmittance ranged from 0.01 to 11.13% for UNT films and from 0.17 to 7.01% for HT ones. Negligible transmittance values were noticed at 200 nm, 250 nm, and 300 nm for both UNT and HT films. A possible explanation for this may be the high content of aromatic amino acids in the protein-based structure that can absorb UV radiation [31]. In the VIS-light domain (400–800 nm), transmittance ranged from 10.10 to 70.65% in UNT films and from 3.72 to 71.98% in HT ones. The above results indicate excellent barrier properties in the UV region for both UNT and HT films and better barrier properties in the VIS region for the HT film.

The addition of tarragon essential oil did not significantly influence the transmittance of film at wavelengths of 200 nm (*p* > 0.05^NS^; contribution of 10.3%) and 250 nm (*p* > 0.05^NS^; contribution of 1.5%); instead, it significantly influenced it at 300 nm (*p* < 0.01 **; contribution of 12.4%), 350 nm (*p* < 0.01 **; contribution of 25.5%), 400 nm (*p* < 0.001 ***; contribution of 37.7%), 450 nm (*p* < 0.001 ***; contribution of 39.9%), 500 nm (*p* < 0.001 ***; contribution of 36.5%), 550 nm (*p* < 0.001 ***; contribution of 36.8%), 600 nm (*p* < 0.001 ***; contribution of 35.5%), 650 nm (*p* < 0.001 ***; contribution of 35.1%), 700 nm (*p* < 0.001 ***; contribution of 35.1%), 750 nm (*p* < 0.001 ***; contribution of 34.8%), and 800 nm (*p* < 0.001 ***; contribution of 34.6%). In the case of UNT film, the transmittance showed an irregular behavior with the addition of tarragon essential oil, displaying an increasing-decreasing-increasing shape. Formulation C showed transmittance values between 0.24% (at 200 nm) and 45.20% (at 800 nm and formulation F2.5 between 0.02% (at 200 nm) and 49.80% (at 800 nm). In the case of HT film, the transmittance value decreased with the addition of tarragon essential oil, thus enhancing its light-barrier properties. Formulation F2.5 had the lowest transmittance values at all measured wavelengths, ranging from 0.30% (at 200 nm) to 7.22% (at 800 nm), and formulation C the highest ones, ranging from 0.42% (at 200 nm) to 71.98% (at 800 nm).

Transparency value was significantly higher (*p* < 0.001 ***) in the HT film, the contribution of heat-treatment factor being 26.3% (see Table S2 in Supplementary Material). Yet, the significant contribution (*p* < 0.001 ***) of the addition of tarragon essential oil to the transparency of film was higher, of 34.7%; an opposite behavior to light transmittance was noticed both in the UNT (irregular) and HT film (ascendant). In UNT films, formulation C had a transparency value of 1.3 (A600/mm) and formulation F2.5 of 0.8 (A600/mm). In HT films, formulation C had a transparency value of 0.5 (A600/mm) while formulation F2.5 of 2.9 (A600/mm). As transmittance values were below 90% in all formulations, both UNT and HT films can be considered semi-transparent.

#### 3.2.8. Puncture Resistance (PR) and Puncture Deformation (PD)

**Figure 9:**
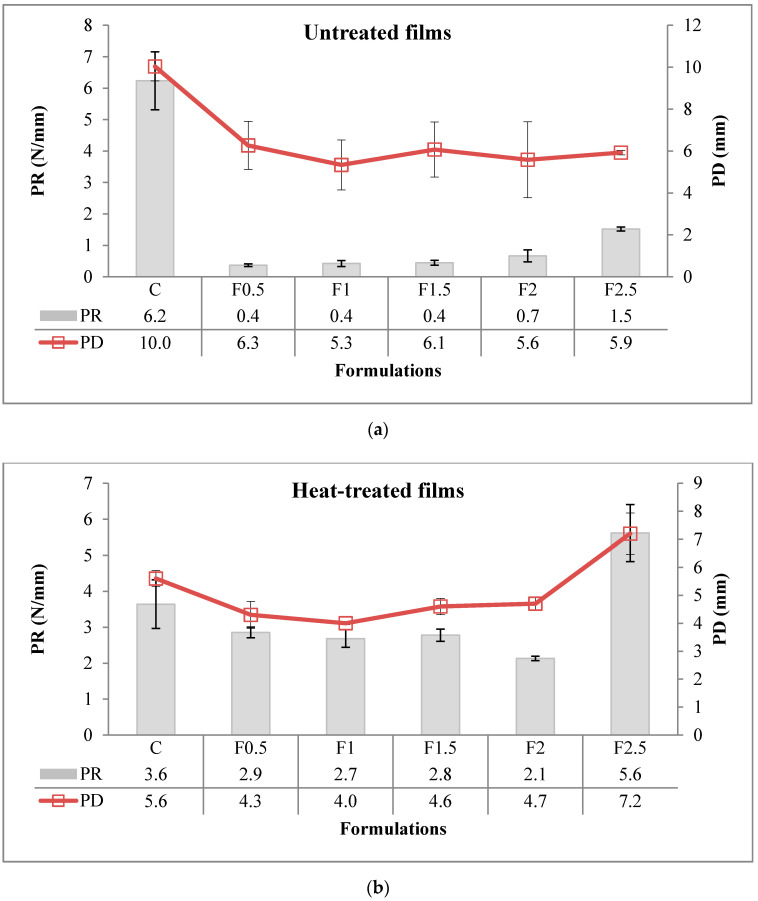
Puncture resistance (PR) and puncture deformation (PD) of edible films. (**a**) Untreated, (**b**) heat-treated. C-control film; F0.5-film with 0.5% tarragon essential oil; F1-film with 1% tarragon essential oil; F1.5-film with 1.5% tarragon essential oil; F2-film with 2% tarragon essential oil; F2.5-film with 2.5% tarragon essential oil. Values are expressed as mean ± standard deviation of four replicates for PR and PD.

PR evaluates the film strength to penetration, while PD, the film elasticity under stress [37,38]. Figure 9a,b shows the values for PR and PD in UNT and HT films. The PR was significantly affected (*p* < 0.001 ***; contribution of 19.0%) by heat treatment of the film-forming solution (see Table S4 in Supplementary Material). The value of PR was higher in HT film, thus showing better resistance to penetration compared to UNT film. The addition of tarragon essential oil also significantly affected (*p* < 0.001 ***) the PR of the film, the contribution of this factor being higher, of 48.6%. In UNT films, PR ranged from 0.4 to 6.2 N/mm manifesting irregular behavior. In HT films, it ranged from 2.1 and 5.6 N/mm, decreasing with the addition of tarragon essential oil up to formulation F2, then increasing in F2.5. Comparable levels of PR, ranging between 3.83 and 4.44 N/mm, were found in edible films prepared from mucilage powders of three cultivars of *Opuntia ficus-indica* (0.5%), pectin (0.7%), glycerol (1%), and (97.8%) water [39]. Higher levels of PR were reported by Jiang et al. (2010) in edible film prepared with 10% WPI, 4% glycerol, and 86% water (of 25.0 N/mm), and by Machado Azevedo et al. (2017) in edible film prepared with 60% corn starch/WPI blend (1:1, *w*/*w*), 24% glycerol, and 16% water (of 11.5 N/mm) [34,40]. These findings would seem to show that PR value is related to the water content of the film. The low values of resistance to puncture force in our films are probably due to their high moisture content.

The PD was significantly affected (*p* < 0.001 ***; contribution of 18.8%) by heat treatment of the film-forming solution (see Table S4 in Supplementary Material). In contrast to PR, the value of PD was lower in HT film, thus showing lower elasticity than UNT film. The contribution of tarragon essential oil addition was also significant (*p* < 0.001 ***) but higher as a share (38.1%). In UNT films, PD varied from 5.3 to 10.0 mm, having chaotic behavior. In HT films, it varied from 4.0 to 7.2 mm, initially decreasing with the addition of tarragon essential oil up to formulation F1, then increasing up to F2.5. This is probably due to the plasticizing effect of tarragon essential oil at a concentration above 1%. Jiang et al. (2010) found a higher level of PD in edible film prepared with 10% WPI, of 12.0 mm [34]. Surprisingly, Machado Azevedo et al. (2017) reported a lower level of PD in edible film prepared with corn starch/WPI blend, of 1.86 mm [40].

## 4. Conclusions

The essential oil of tarragon has shown to possess both antioxidant and antibacterial activities. The WPI-based edible film was affected by heat treatment of the filmogenic solution. HT film showed improved physical and mechanical properties, being more transparent, less soluble in water, more light protective in the range of 350–800 nm, and more resistant to mechanical penetration. Therefore, it is more suitable for certain end-use applications. With the addition of tarragon essential oil, the HT film became more red-yellowish, more transparent, more protective against VIS light, more resistant to puncture, and more elastic. Further research is needed to determine the effectiveness of the edible film with 2.5% tarragon essential oil in extending the shelf-life of food.

## Figures and Tables

**Table 1:** Retention times (min) and relative contents (%) of volatile constituents identified in tarragon essential oil.

Crt. No.	Compound	Chemical Class	Retention Time	Relative Content
1	Hexanal	A.A.	5.451	0.18
2	**α-Phellandrene**	M.C.	9.695	**2.27**
3	α-Pinene	M.C.	9.959	1.35
4	**Sabinene**	M.C.	11.537	**74.98**
5	β-Pinene	M.C.	11.709	1.76
6	**β-Myrcene**	M.C.	12.224	**3.38**
7	**4-Carene**	M.C.	13.306	**2.58**
8	*o*-Cymene	M.C.	13.627	1.10
9	**D-Limonene**	M.C.	13.806	**3.69**
10	1,8-cineole	O.M.	13.947	0.10
11	trans-β-Ocimene	M.C.	14.119	0.70
12	cis-β-Ocimene	M.C.	14.538	0.36
13	**γ-Terpinene**	M.C.	14.990	**3.80**
14	Terpinolene	M.C.	16.083	0.69
15	trans-4-Thujanol	O.M.	19.882	0.89
16	Isoeugenol methyl ether	Phe.P.	27.898	2.18
-	TOTAL	-	-	100.00

A.A.—aliphatic aldehyde; M.C.—monoterpene hydrocarbon; O.M.—oxygenated monoterpene; Phe.P.—phenylpropanoid.

**Table 2:** Diameters of inhibition zones (mm) of bacterial strains produced by the tarragon essential oil and gentamicin.

Bacterial Strain	Tarragon Essential Oil	Gentamicin
*E. coli* (ATCC 25922)	12.03 ± 0.47	25.27 ± 0.08
*S. enteritidis* (ATCC 13076)	14.41 ± 0.29	23.39 ± 0.03
*S. aureus* (ATCC 25923)	13.53 ± 0.38	25.77 ± 0.39
*L. monocytogenes* (ATCC 19114)	11.42 ± 0.27	26.67 ± 1.68

Values are expressed as mean ± standard deviation of three replicates.

**Table 3:** Minimum inhibitory concentration (MIC, µL EO mL^−1^) and minimum bactericidal concentration (MBC, µL EO mL^−1^) of tarragon essential oil.

Bacterial Strain	Tarragon Essential Oil	
	MIC	MBC
*E. coli* (ATCC 25922)	5.14 ± 0.0	5.14 ± 0.0
*S. enteritidis* (ATCC 13076)	10.80 ± 0.0	10.80 ± 0.0
*S. aureus* (ATCC 25923)	10.80 ± 0.0	22.68 ± 0.0
*L. monocytogenes* (ATCC 19114)	5.14 ± 0.0	10.80 ± 0.0

Values are expressed as mean ± standard deviation of three replicates. The MICs of gentamicin for *E. coli* (ATCC 25922), *S. enteritidis* (ATCC 13076), *S. aureus* (ATCC 25923), and *L. monocytogenes* (ATCC 19114) were 0.50, 0.11, 0.05, and 0.05 µg GE mL^−1^, respectively.

## Supplementary Materials

The following are available online at https://www.mdpi.com/2073-4360/12/8/1748/s1, Table S1: Effects of heat treatment of the film-forming solution, the addition of tarragon essential oil, and their first-degree interaction on thickness, moisture content, swelling degree, solubility in water, and WVP of edible films. Table S2: Effects of heat treatment of the film-forming solution, the addition of tarragon essential oil, and their first-degree interaction on color and transparency of edible films. Table S3: Effects of heat treatment of the film-forming solution, the addition of tarragon essential oil, and their first-degree interaction on light transmittance of edible films. Table S4: Effects of heat treatment of the film-forming solution, the addition of tarragon essential oil, and their first-degree interaction on puncture resistance and puncture deformation of edible films.
