# Supplementary material for: Formulation and Characterization of Antimicrobial Edible Films Based on Whey Protein Isolate and Tarragon Essential Oil
Source: Polymers (Basel). 2020 Aug 5;12(8):1748. doi: 10.3390/polym12081748 (PMC7464654; doi:10.3390/polym12081748)
Supplement: Supplementary file 1 [file polymers-12-01748-s001.pdf]

Table S1. Effects of heat-treatment of the film-forming solution, the addition of tarragon essential oil, and their first-degree interaction on thickness, moisture content, swelling degree, solubility in water, and WVP of edible films.

Factor	Thickness (mm)	Moisture Content (%)	Swelling Degree (%)	Solubility in Water (%)	WVP (g mm/m2 day kPa)
Heat-treatment					
UNT	0.43^{b}	45.8^{b}	100.5^{b}	97.5^{a}	7.6^{b}
HT	0.46^{a}	52.9^{a}	101.4^{a}	96.7^{b}	8.5^{a}
p/CTR (%)	0.000^{***}/21.8	0.000^{***}/45.2	0.000^{***}/68.1	0.000^{***}/14.5	0.003^{**}/19.9
Addition of tarragon essential oil					
C	0.45^{a}	46.2^{c}	100.8^{ab}	95.3^{a}	7.5^{a}
F0.5	0.45^{a}	46.3^{c}	101.1^{a}	96.8^{c}	7.4^{a}
F1	0.44^{a}	48.8^{bc}	101.1^{a}	97.1^{bc}	8.0^{a}
F1.5	0.45^{a}	50.6^{ab}	100.6^{b}	97.3^{b}	8.2^{a}
F2	0.44^{a}	51.4^{ab}	101.0^{ab}	97.9^{a}	8.7^{a}
F2.5	0.44^{a}	53.0^{a}	100.9^{ab}	98.0^{a}	8.5^{a}
p/CTR (%)	0.606^{NS}/1.0	0.000^{***}/23.7	0.005^{**}/9.2	0.000^{***}/68.7	0.026^{*}/27.8
Heat-treatment x Addition of tarragon essential oil					
UNT x C	0.44 ± 0.02^{abc}	44.2 ± 1.81^{ef}	100.5 ± 0.07^{ef}	95.6 ± 0.18^{e}	7.7 ± 0.14^{abc}
UNT x F0.5	0.44 ± 0.01^{bc}	40.4 ± 7.12^{f}	100.4 ± 0.01^{f}	96.7 ± 0.15^{d}	6.7 ± 0.50^{c}
UNT x F1	0.43 ± 0.02^{c}	44.1 ± 2.24^{ef}	100.4 ± 0.04^{f}	97.1 ± 0.09^{cd}	6.9 ± 0.07^{c}
UNT x F1.5	0.43 ± 0.02^{c}	49.4 ± 1.12^{bcde}	100.4 ± 0.09^{f}	97.9 ± 0.12^{bc}	8.3 ± 0.35^{abc}
HT x F2	0.43 ± 0.01^{c}	46.4 ± 2.75^{def}	100.4 ± 0.11^{f}	98.9 ± 0.12^{a}	7.8 ± 0.14^{abc}
UNT x F2.5	0.43 ± 0.02^{c}	50.6 ± 2.06^{abcde}	100.6 ± 0.04^{def}	98.6 ± 0.21^{ab}	8.7 ± 0.07^{abc}
HT x C	0.45 ± 0.03^{ab}	48.1 ± 0.62^{cde}	101.1 ± 0.03^{cde}	95.0 ± 0.37^{e}	7.3 ± 0.50^{bc}
HT x F0.5	0.45 ± 0.02^{ab}	52.1 ± 1.89^{abcd}	101.7 ± 0.08^{ab}	96.8 ± 0.17^{d}	8.2 ± 1.20^{abc}
HT x F1	0.46 ± 0.02^{ab}	53.5 ± 2.03^{abc}	101.9 ± 0.23^{a}	97.1 ± 0.07^{cd}	9.3 ± 0.64^{ab}
HT x F1.5	0.46 ± 0.03^{a}	51.8 ± 0.80^{abcd}	100.9 ± 0.17^{def}	96.8 ± 0.50^{d}	8.2 ± 0.71^{abc}
HT x F2	0.46 ± 0.02^{ab}	56.4 ± 1.12^{a}	101.6 ± 0.61^{abc}	96.8 ± 0.47^{d}	9.6 ± 0.71^{a}
HT x F2.5	0.46 ± 0.02^{ab}	55.5 ± 2.78^{ab}	101.2 ± 0.29^{bcd}	97.4 ± 0.42^{cd}	8.5 ± 0.35^{abc}
p/CTR (%)	0.044^{*}/3.1	0.007^{**}/10.9	0.001^{**}/12.8	0.000^{***}/12.3	0.012^{*}/35.0

UNT-untreated films; HT-heat-treated films; C-control film; F0.5-film with 0.5% tarragon essential oil; F1-film with 1% tarragon essential oil; F1.5-film with 1.5% tarragon essential oil; F2-film with 2% tarragon essential oil; F2.5-film with 2.5% tarragon essential oil; WVP-water vapor permeability; *p*-probability; CTR (%)-percentage contribution. Values are expressed as mean ± standard deviation of twenty-four replicates for thickness, four for moisture content, respectively three replicates for swelling degree, solubility in water, and WVP. Different letters in the same column indicate statistically significant differences at $p < 0.05$ (Tukey's test). Significance: $p \geq 0.05^{NS}$, not significant; $p < 0.05^{*}$, significant; $p < 0.01^{**}$, very significant; $p < 0.001^{***}$, extremely significant.

Table S2. Effects of heat-treatment of the film-forming solution, the addition of tarragon essential oil, and their first-degree interaction on color parameters and transparency of edible films.

Factor	Color Parameters				Transparency (A600/mm)
	*L**	*a**	*b**	*ΔE**	
Heat-treatment					
UNT	81.27^{a}	2.80^{a}	18.05^{b}	23.06^{b}	0.9^{b}
HT	79.51^{b}	2.12^{b}	20.72^{a}	26.13^{a}	1.8^{a}
p/CTR (%)	0.000***/27.5	0.000***/13.3	0.000***/16.6	0.000***/20.2	0.000***/26.3
Addition of tarragon essential oil					
C	81.98^{a}	1.77^{d}	15.38^{d}	20.30^{d}	0.9^{b}
F0.5	80.33^{c}	2.03^{cd}	18.27^{c}	23.55^{c}	0.6^{b}
F1	81.12^{b}	1.81^{d}	18.05^{c}	23.05^{c}	1.1^{b}
F1.5	80.13^{cd}	2.29^{c}	19.39^{b}	24.65^{b}	1.7^{a}
F2	79.57^{de}	3.17^{b}	22.47^{a}	27.79^{a}	2.0^{a}
F2.5	79.22^{e}	3.68^{a}	22.75^{a}	28.22^{a}	1.9^{a}
p/CTR (%)	0.000***/30.6	0.000***/60.4	0.000***/61.9	0.000***/65.0	0.000***/34.7
Heat-treatment x Addition of tarragon essential oil					
UNT x C	84.46±0.89^{a}	1.66±0.70^{f}	14.75±0.96^{g}	18.67±1.07^{f}	1.3±0.50^{cd}
UNT x F0.5	81.11±0.68^{b}	2.49±0.31^{cd}	16.13±1.04^{f}	21.33±1.16^{d}	0.5±0.06^{cd}
UNT x F1	80.91±0.65^{b}	2.06±0.30^{cdef}	14.41±0.67^{g}	19.87±0.62^{e}	0.6±0.13^{cd}
UNT x F1.5	80.54±0.49^{bcd}	2.60±0.15^{c}	17.52±0.55^{g}	22.83±0.52^{c}	1.0±0.10^{cd}
HT x F2	80.70±1.09^{bc}	3.95±0.83^{c}	22.61±0.30^{ab}	27.57±0.56^{a}	1.2±0.28^{cd}
UNT x F2.5	79.90±0.53^{cde}	4.02±0.22^{c}	22.89±0.32^{a}	28.11±0.34^{a}	0.8±0.23^{cd}
HT x C	79.50±0.38^{ef}	1.88±0.29^{ef}	16.01±0.21^{f}	21.93±0.31^{cd}	0.5±0.08^{d}
HT x F0.5	79.54±1.19^{de}	1.57±0.21^{f}	20.41±1.13^{d}	25.78±1.07^{b}	0.8±0.08^{cd}
HT x F1	81.34±1.03^{b}	1.55±0.42^{f}	21.69±1.18^{bc}	26.22±1.36^{b}	1.5±0.25^{bc}
HT x F1.5	79.72±0.47^{cde}	1.97±0.27^{def}	21.26±0.47^{cd}	26.48±0.39^{b}	2.5±0.78^{ab}
HT x F2	78.45±0.42^{g}	2.40±0.24^{cde}	22.34±0.37^{ab}	28.02±0.39^{a}	2.8±0.71^{a}
HT x F2.5	78.53±0.33^{fg}	3.35±0.45^{b}	22.62±0.41^{ab}	28.34±0.40^{a}	2.9±0.07^{a}
p/CTR (%)	0.000***/24.2	0.000***/8.0	0.000***/17.1	0.000***/10.0	0.000***/28.0

UNT-untreated films; HT-heat-treated films; C-control film; F0.5-film with 0.5% tarragon essential oil; F1-film with 1% tarragon essential oil; F1.5-film with 1.5% tarragon essential oil; F2-film with 2% tarragon essential oil; F2.5-film with 2.5% tarragon essential oil; p-probability; CTR (%)-percentage contribution. Values are expressed as mean ± standard deviation of twelve replicates for color parameters and tree replicates for transparency. Different letters in the same column indicate statistically significant differences at $p < 0.05$ (Tukey's test). Significance: $p < 0.001$***, extremely significant.

Table S3. Effects of heat-treatment of the film-forming solution, the addition of tarragon essential oil, and their first-degree interaction on light transmittance of edible films.

Factor	Light Transmittance (%)												
	**at 200 nm**	**at 250 nm**	**at 300 nm**	**at 350 nm**	**at 400 nm**	**at 450 nm**	**at 500 nm**	**at 550 nm**	**at 600 nm**	**at 650 nm**	**at 700 nm**	**at 750 nm**	**at 800 nm**
Heat-treatment													
UNT	0.06^{b}	0.04^{b}	0.07^{b}	6.23^{a}	25.37^{a}	36.89^{a}	44.19^{a}	48.88^{a}	51.66^{a}	53.21^{a}	54.29^{a}	54.88^{a}	55.34^{a}
HT	0.35^{a}	0.27^{a}	0.27^{a}	2.77^{b}	12.83^{b}	20.02^{b}	24.29^{b}	27.07^{b}	28.01^{b}	28.98^{b}	29.71^{b}	30.11^{b}	30.53^{b}
p/CTR (%)	0.000***/66.6	0.000***/71.9	0.000***/58.4	0.000***/19.2	0.000***/28.0	0.000***/28.1	0.000***/28.1	0.000***/28.8	0.000***/30.1	0.000***/30.1	0.000***/30.3	0.000***/30.0	0.000***/29.7
Addition of tarragon essential oil													
C	0.33^{a}	0.15^{a}	0.26^{a}	7.74^{a}	28.73^{a}	41.35^{a}	47.43^{ab}	51.83^{ab}	54.55^{a}	56.04^{a}	57.15^{a}	57.87^{a}	58.59^{a}
F0.5	0.18^{ab}	0.15^{a}	0.14^{b}	4.93^{a}	27.18^{ab}	41.35^{a}	50.24^{a}	55.63^{a}	58.69^{a}	60.34^{a}	61.44^{a}	61.94^{a}	62.17^{a}
F1	0.20^{ab}	0.18^{a}	0.19^{ab}	4.10^{ab}	18.76^{bc}	28.40^{b}	35.15^{bc}	40.13^{bc}	40.73^{b}	42.27^{b}	43.50^{b}	43.99^{b}	44.52^{b}
F1.5	0.19^{ab}	0.16^{a}	0.16^{ab}	4.46^{ab}	16.76^{c}	24.06^{bc}	28.35^{cd}	30.58^{cd}	31.90^{bc}	32.64^{bc}	33.12^{bc}	33.42^{bc}	33.70^{bc}
F2	0.19^{ab}	0.15^{a}	0.15^{b}	0.90^{b}	7.08^{d}	14.09^{c}	19.68^{d}	23.54^{d}	26.01^{c}	27.57^{c}	28.71^{c}	29.48^{c}	30.14^{c}
F2.5	0.16^{b}	0.13^{a}	0.12^{b}	4.89^{a}	16.06^{c}	21.49^{bc}	24.58^{cd}	26.13^{d}	27.12^{c}	27.71^{c}	28.08^{c}	28.29^{c}	28.51^{c}
p/CTR (%)	0.058^{NS}/10.3	0.727^{NS}/1.5	0.007**/12.4	0.001**/25.5	0.000***/37.7	0.000***/39.9	0.000***/36.5	0.000***/36.8	0.000***/35.5	0.000***/35.1	0.000***/35.1	0.000***/34.8	0.000***/34.6
Heat-treatment x Addition of tarragon essential oil													
UNT x C	0.24±0.26^{abc}	0.14±0.07^{bc}	0.26±0.13^{a}	11.13±3.95^{a}	29.83±9.98^{a}	37.85±12.16^{ab}	39.21±17.10^{abc}	41.86±17.36^{abc}	43.45±17.03^{bc}	44.02±17.09^{bc}	44.53±16.91^{bc}	44.91±16.67^{cd}	45.20±16.52^{cd}
UNT x F0.5	0.06±0.08^{bc}	0.04±0.06^{c}	0.05±0.04^{bc}	2.86±1.28^{cde}	26.38±5.61^{a}	42.80±6.34^{a}	54.60±6.03^{a}	62.12±5.67^{a}	66.27±5.34^{a}	68.54±5.35^{a}	70.02±5.37^{a}	70.61±5.38^{ab}	70.65±5.10^{ab}
UNT x F1	0.01±0.0^{c}	0.01±0.0^{c}	0.03±0.0^{c}	4.90±1.19^{abcde}	25.76±1.17^{a}	39.31±4.31^{a}	49.49±4.98^{ab}	55.44±5.51^{ab}	58.85±5.72^{ab}	60.81±5.70^{ab}	62.17±5.68^{ab}	62.88±5.61^{abc}	63.70±5.53^{abc}
UNT x F1.5	0.02±0.02^{bc}	0.02±0.02^{c}	0.03±0.01^{c}	8.26±1.46^{abc}	29.79±0.88^{a}	40.75±1.13^{a}	46.94±1.91^{ab}	50.01±2.49^{ab}	51.89±2.75^{ab}	52.97±2.68^{ab}	53.67±2.72^{ab}	54.09±2.87^{abc}	54.41±2.77^{abc}
HT x F2	0.02±0.01^{bc}	0.02±0.01^{c}	0.04±0.01^{c}	0.85±0.42^{de}	10.10±2.32^{b}	21.42±3.00^{bc}	30.81±3.92^{bc}	37.40±5.08^{bc}	41.54±5.96^{bc}	44.11±6.66^{bc}	46.03±7.12^{bc}	47.27±7.51^{c}	48.30±7.77^{c}
UNT x F2.5	0.02±0.01^{bc}	0.02±0.01^{c}	0.03±0.01^{c}	9.39±3.75^{ab}	30.34±6.88^{a}	39.21±7.93^{a}	44.06±8.29^{ab}	46.45±8.62^{ab}	47.94±8.65^{ab}	48.81±8.58^{ab}	49.31±8.45^{ab}	49.56±8.33^{bc}	49.80±8.21^{bc}
HT x C	0.42±0.15^{a}	0.17±0.10^{bc}	0.26±0.03^{a}	4.34±1.09^{bcde}	27.64±3.17^{a}	44.84±3.82^{a}	55.65±4.13^{a}	61.80±4.23^{c}	65.66±4.34^{a}	68.06±4.15^{a}	69.75±4.18^{a}	70.83±4.44^{a}	71.98±4.42^{a}
HT x F0.5	0.30±0.03^{ab}	0.25±0.05^{ab}	0.23±0.03^{a}	7.01±3.57^{abcd}	27.98±4.21^{a}	39.90±1.71^{a}	45.89±0.16^{ab}	49.14±1.06^{ab}	51.10±1.51^{ab}	52.14±1.68^{ab}	52.86±1.71^{ab}	53.27±1.69^{abc}	53.68±1.77^{abc}
HT x F1	0.39±0.07^{a}	0.36±0.09^{a}	0.35±0.08^{a}	3.30±2.32^{bcde}	11.77±5.12^{b}	17.49±5.23^{cd}	20.82±4.93^{cd}	24.82±3.58^{cd}	22.61±4.49^{cd}	23.74±4.15^{cd}	24.82±3.58^{cd}	25.10±3.36^{de}	25.34±3.22^{de}
HT x F1.5	0.35±0.04^{a}	0.31±0.04^{ab}	0.28±0.04^{a}	0.65±0.53^{e}	3.72±4.30^{b}	7.37±7.44^{cd}	9.75±9.00^{d}	11.14±9.74^{d}	11.92±9.99^{d}	12.31±9.93^{d}	12.57±9.76^{d}	12.75±9.65^{e}	12.99±9.68^{e}
HT x F2	0.35±0.08^{a}	0.29±0.10^{ab}	0.26±0.10^{a}	0.96±0.81^{de}	4.07±3.31^{b}	6.77±4.63^{cd}	8.56±5.02^{d}	9.68±4.95^{d}	10.47±4.79^{d}	11.03±4.55^{d}	11.40±4.21^{d}	11.69±3.89^{e}	11.98±3.56^{e}
HT x F2.5	0.30±0.05^{ab}	0.24±0.03^{ab}	0.22±0.05^{ab}	0.38±0.15^{e}	1.79±0.77^{b}	3.77±1.13^{d}	5.13±1.71^{d}	5.09±1.21^{d}	6.29±1.19^{d}	6.60±1.21^{d}	6.86±1.26^{d}	7.03±1.26^{e}	7.22±1.27^{e}
p/CTR (%)	0.532^{NS}/3.5	0.003**/13.5	0.003**/15.0	0.000***/19.7	0.000***/23.5	0.000***/23.2	0.000***/26.0	0.000***/26.0	0.000***/26.8	0.000***/27.5	0.000***/28.0	0.000***/28.5	0.000***/29.2

UNT-untreated films; HT-heat-treated films; C-control film; F0.5-film with 0.5% tarragon essential oil; F1-film with 1% tarragon essential oil; F1.5-film with 1.5% tarragon essential oil; F2-film with 2% tarragon essential oil; F2.5-film with 2.5% tarragon essential oil; *p*-probability; CTR (%)-percentage contribution. Values are expressed as mean ± standard deviation of three replicates. Different letters in the same column indicate statistically significant differences at $p < 0.05$ (Tukey's test). Significance: $p \geq 0.05^{NS}$, not significant; $p < 0.01$**, very significant; $p < 0.001$***, extremely significant.

Table S4. Effects of heat-treatment of the film-forming solution, the addition of tarragon essential oil, and their first-degree interaction on puncture resistance and puncture deformation of edible films.

Factor	Puncture Resistance (N/mm)	Puncture Deformation (mm)
Heat-treatment		
UNT	1.6^{b}	6.5^{a}
HT	3.3^{a}	5.1^{b}
p/CTR (%)	0.000***/19.0	0.000***/18.8
Addition of tarragon essential oil		
C	4.9^{a}	7.8^{a}
F0.5	1.6^{c}	5.3^{bc}
F1	1.6^{c}	4.6^{c}
F1.5	1.6^{c}	5.3^{bc}
F2	1.4^{c}	5.1^{c}
F2.5	3.6^{b}	6.6^{ab}
p/CTR (%)	0.000***/48.6	0.000***/38.1
Heat-treatment x Addition of tarragon essential oil		
UNT x C	6.2±0.921^{a}	10.0±0.684^{a}
UNT x F0.5	0.4±0.041^{f}	6.3±1.149^{bc}
UNT x F1	0.4±0.098^{f}	5.3±1.190^{bcd}
UNT x F1.5	0.4±0.078^{f}	6.1±1.312^{bcd}
HT x F2	0.7±0.190^{ef}	5.6±1.809^{bcd}
UNT x F2.5	1.5±0.064^{de}	5.9±0.109^{bcd}
HT x C	3.6±0.675^{b}	5.6±0.291^{bcd}
HT x F0.5	2.9±0.148^{bc}	4.3±0.484^{cd}
HT x F1	2.7±0.244^{bc}	4.0±0.051^{d}
HT x F1.5	2.8±0.169^{bc}	4.6±0.289^{cd}
HT x F2	2.1±0.064^{cd}	4.7±0.218^{cd}
HT x F2.5	5.6±0.791^{a}	7.2±0.737^{b}
p/CTR (%)	0.000***/28.8	0.000***/24.1

UNT-untreated films; HT-heat-treated films; C-control film; F0.5-film with 0.5% tarragon essential oil; F1-film with 1% tarragon essential oil; F1.5-film with 1.5% tarragon essential oil; F2-film with 2% tarragon essential oil; F2.5-film with 2.5% tarragon essential oil; p-probability; CTR (%)-percentage contribution. Values are expressed as mean ± standard deviation of four replicates. Different letters in the same column indicate statistically significant differences at $p < 0.05$ (Tukey's test). Significance: $p < 0.001$***, extremely significant.
